# Supplementary material for: Exploring the drivers of price variation in orthopaedic radical bone tumor resection: A nationwide database study
Source: PLoS One. 2026 Feb 26;21(2):e0343676. doi: 10.1371/journal.pone.0343676 (PMC12944713; doi:10.1371/journal.pone.0343676)
Supplement: S1 Table — (DOCX) [file pone.0343676.s001.docx]

**Table S1: State Divisions According to United States Census Bureau^[[1]](#footnote-1)^**

| **United States Census Bureau Region** | **Included States** |
| --- | --- |
| New England | 1. Connecticut 2. Maine 3. Massachusetts 4. New Hampshire 5. Rhode Island 6. Vermont |
| Mid-Atlantic | 1. New Jersey 2. New York 3. Pennsylvania |
| East North Central | 1. Illinois 2. Indiana 3. Michigan 4. Ohio 5. Wisconsin |
| West North Central | 1. Iowa 2. Kansas 3. Minnesota 4. Missouri 5. Nebraska 6. North Dakota 7. South Dakota |
| South Atlantic | 1. Delaware 2. District of Columbia 3. Florida 4. Georgia 5. Maryland 6. North Carolina 7. South Carolina 8. Virginia 9. West Virginia |
| East South Central | 1. Alabama 2. Kentucky 3. Mississippi 4. Tennessee |
| West South Central | 1. Arkansas 2. Louisiana 3. Oklahoma 4. Texas |
| Mountain | 1. Arizona 2. Colorado 3. Idaho 4. Montana 5. Nevada 6. New Mexico 7. Utah 8. Wyoming |
| Pacific | 1. Alaska 2. California 3. Hawaii 4. Oregon 5. Washington |

1. https://www2.census.gov/geo/pdfs/maps-data/maps/reference/us_regdiv.pdf [↑](#footnote-ref-1)
