# Supplementary material for: Exploring the drivers of price variation in orthopaedic radical bone tumor resection: A nationwide database study
Source: PLoS One. 2026 Feb 26;21(2):e0343676. doi: 10.1371/journal.pone.0343676 (PMC12944713; doi:10.1371/journal.pone.0343676)
Supplement: S2 Table — (DOCX) [file pone.0343676.s002.docx]

**Table S2: Multivariable Linear Regression for Payor Rates within the Radical Resection of Humerus Cohort**

| Variable | Estimate (USD)^a^ | p-value | Lower Limit, 95% Confidence Interval (USD) | Upper Limit, 95% Confidence Interval (USD) |
| --- | --- | --- | --- | --- |
| *Total Bed Range of Hospital* | | | | |
| 1 - 100 | Reference | Reference | Reference | Reference |
| 100 - 300 | $163.47 | <0.001* | $130.68 | $196.25 |
| 300 - 500 | $-34.95 | 0.0685 | $-72.54 | $2.65 |
| 500 - 1000 | $-98.48 | <0.001* | $-140.38 | $-56.58 |
| 1000 - 1500 | $469.45 | <0.001* | $348.82 | $590.08 |
| 1500 + | $797.46 | <0.001* | $585.88 | $1009.03 |
| *Payor Class* | | | | |
| Commercial | Reference | Reference | Reference | Reference |
| Dual | $-14.97 | 0.83 | $-155.26 | $125.31 |
| Managed Medicaid | $-2016.69 | <0.001* | $-2060.35 | $-1973.03 |
| Medicare Advantage | $71.18 | <0.001* | $39.68 | $102.68 |
| Veterans Affairs | $-295.97 | <0.001* | $-389.10 | $-202.84 |
| Workers' Compensation | $1543.66 | <0.001* | $1443.41 | $1643.90 |
| *Hospital Type* | | | | |
| Acute Care | Reference | Reference | Reference | Reference |
| Critical Access | $-703.76 | <0.001* | $-764.73 | $-642.79 |
| *U.S. Census Bureau Division* | | | | |
| Middle Atlantic | Reference | Reference | Reference | Reference |
| New England | $1769.43 | <0.001* | $1563.83 | $1975.03 |
| East North Central | $-983.21 | <0.001* | $-1059.54 | $-906.88 |
| East South Central | $172.04 | 0.002* | $63.93 | $280.15 |
| Mountain | $197.18 | <0.001* | $117.13 | $277.23 |
| Pacific | $1576.57 | <0.001* | $1485.99 | $1667.15 |
| South Atlantic | $1143.36 | <0.001* | $1041.10 | $1245.63 |
| West North Central | $625.83 | <0.001* | $545.56 | $706.10 |
| West South Central | $313.16 | <0.001* | $236.22 | $390.10 |
| Abbreviations: USD = United States Dollars  ^a^ A “-” symbol preceding the estimate corresponds to a reduction in payor rates in comparison to the reference group.  *Statistically significant, p < 0.05  R^2^ = 0.24 | | | | |
