# Supplementary material for: Exploring the drivers of price variation in orthopaedic radical bone tumor resection: A nationwide database study
Source: PLoS One. 2026 Feb 26;21(2):e0343676. doi: 10.1371/journal.pone.0343676 (PMC12944713; doi:10.1371/journal.pone.0343676)
Supplement: S3 Table — (DOCX) [file pone.0343676.s003.docx]

**Table S3: Multivariable Linear Regression for Payor Rates within the Radical Resection of Humerus Cohort, Health Policy Sub-Analysis**

| Health Policy Variable^a^ | Estimate (USD)^b^ | p-value | Lower Limit, 95% Confidence Interval (USD) | Upper Limit, 95% Confidence Interval (USD) |
| --- | --- | --- | --- | --- |
| Medicaid Expansion Status | | | | |
| No | Reference | Reference | Reference | Reference |
| Yes | $274.64 | <0.001* | $214.64 | $334.64 |
| Certificate of Need Status | | | | |
| No | Reference | Reference | Reference | Reference |
| Yes | $348.25 | <0.001* | $288.25 | $408.26 |
| Scope of Independent Practice Regulations for Nurse Practitioners | | | | |
| No Practice | Reference | Reference | Reference | Reference |
| Restricted Practice | $662.08 | <0.001* | $604.24 | $719.92 |
| Full Practice | $-226.29 | <0.001* | $-305.22 | $-147.35 |
| All Payor-Claims (i.e. Price Transparency) Database Mandate | | | | |
| No | Reference | Reference | Reference | Reference |
| Yes | $0.78 | 0.97 | $-34.24 | $35.80 |
| Abbreviations: USD = United States Dollars  ^a^These are state-level variables and denote whether the negotiated payor rate was found within a state that implemented each respective policy.  ^b^A “-” symbol preceding the estimate corresponds to a reduction in payor rates in comparison to the reference group.  *Statistically significant, p < 0.05  R^2^ = 0.19 | | | | |
