# Supplementary material for: Exploring the drivers of price variation in orthopaedic radical bone tumor resection: A nationwide database study
Source: PLoS One. 2026 Feb 26;21(2):e0343676. doi: 10.1371/journal.pone.0343676 (PMC12944713; doi:10.1371/journal.pone.0343676)
Supplement: S4 Table — (DOCX) [file pone.0343676.s004.docx]

**Table S4: Multivariable Linear Regression Results for Payor Rates within the Radical Resection of Femur/Knee Cohort**

| Variable | Estimate (USD)^a^ | p-value | Lower Limit, 95% Confidence Interval (USD) | Upper Limit, 95% Confidence Interval (USD) |
| --- | --- | --- | --- | --- |
| *Total Bed Range of Hospital* | | | | |
| 1 - 100 | Reference | Reference | Reference | Reference |
| 100 - 300 | $-236.27 | <0.001* | $-292.93 | $-179.60 |
| 300 - 500 | $-441.46 | <0.001* | $-502.86 | $-380.07 |
| 500 - 1000 | $-296.51 | <0.001* | $-368.39 | $-224.62 |
| 1000 - 1500 | $1753.12 | <0.001* | $1561.12 | $1945.11 |
| 1500 + | $3409.96 | <0.001* | $3165.47 | $3654.45 |
| *Payor Class* | | | | |
| Commercial | Reference | Reference | Reference | Reference |
| Dual | $-2218.99 | <0.001* | $-2611.39 | $-1826.60 |
| Managed Medicaid | $-2082.38 | <0.001* | $-2142.15 | $-2022.61 |
| Medicare Advantage | $-2946.11 | <0.001* | $-3007.68 | $-2884.53 |
| Veterans Affairs | $-2156.83 | <0.001* | $-2387.21 | $-1926.45 |
| Workers' Compensation | $-375.72 | <0.001* | $-557.31 | $-194.13 |
| *Hospital Type* | | | | |
| Acute Care | Reference | Reference | Reference | Reference |
| Critical Access | -$1073.85 | <0.001* | -$1149.93 | -$997.76 |
| *U.S. Census Bureau Division* | | | | |
| Middle Atlantic | Reference | Reference | Reference | Reference |
| New England | $2204.43 | <0.001* | $2010.22 | $2398.63 |
| East North Central | $2264.97 | <0.001* | $2139.26 | $2390.68 |
| East South Central | $2730.81 | <0.001* | $2557.84 | $2903.79 |
| Mountain | $2203.69 | <0.001* | $2098.30 | $2309.08 |
| Pacific | $271.28 | <0.001* | $171.36 | $371.21 |
| South Atlantic | $3791.18 | <0.001* | $3638.89 | $3943.48 |
| West North Central | $3143.80 | <0.001* | $3050.50 | $3237.09 |
| West South Central | $252.58 | <0.001* | $129.03 | $376.13 |
| Abbreviations: USD = United States Dollars  ^a^ A “-” symbol preceding the estimate corresponds to a reduction in payor rates in comparison to the reference group.  *Statistically significant, p < 0.05  R^2^ = 0.22 | | | | |
