# Supplementary material for: Exploring the drivers of price variation in orthopaedic radical bone tumor resection: A nationwide database study
Source: PLoS One. 2026 Feb 26;21(2):e0343676. doi: 10.1371/journal.pone.0343676 (PMC12944713; doi:10.1371/journal.pone.0343676)
Supplement: S5 Table — (DOCX) [file pone.0343676.s005.docx]

**Table S5: Multivariable Linear Regression Results for Radical Resection of Femur/Knee Cohort, Health Policy Sub-Analysis**

| Health Policy Variable^a^ | Estimate (USD)^b^ | p-value | Lower Limit, 95% Confidence Interval (USD) | Upper Limit, 95% Confidence Interval (USD) |
| --- | --- | --- | --- | --- |
| Medicaid Expansion Status | | | | |
| No | Reference | Reference | Reference | Reference |
| Yes | $1399.41 | <0.001* | $1310.93 | $1487.89 |
| Certificate of Need Status | | | | |
| No | Reference | Reference | Reference | Reference |
| Yes | $667.98 | <0.001* | $578.80 | $757.15 |
| Scope of Independent Practice Regulations for Nurse Practitioners | | | | |
| No Practice | Reference | Reference | Reference | Reference |
| Restricted Practice | $1563.49 | <0.001* | $1477.68 | $1649.30 |
| Full Practice | $-250.79 | <0.001* | $-362.50 | $-139.07 |
| All Payor-Claims (i.e. Price Transparency) Database Mandate | | | | |
| No | Reference | Reference | Reference | Reference |
| Yes | $1231.24 | <0.001* | $1174.38 | $1288.09 |
| Abbreviations: USD = United States Dollars  ^a^These are state-level variables and denote whether the negotiated payor rate was found within a state that implemented each respective policy.  ^b^A “-” symbol preceding the estimate corresponds to a reduction in payor rates in comparison to the reference group.  *Statistically significant, p < 0.05  R^2^ = 0.19 | | | | |
