# Supplementary material for: Exploring the drivers of price variation in orthopaedic radical bone tumor resection: A nationwide database study
Source: PLoS One. 2026 Feb 26;21(2):e0343676. doi: 10.1371/journal.pone.0343676 (PMC12944713; doi:10.1371/journal.pone.0343676)
Supplement: Attachment — Submitted filename: Response to Reviewers.docx [file pone.0343676.s008.docx]

**PONE-D-25-44873**

**Exploring the Drivers of Price Variation in Radical Bone Tumor Resection: A Nationwide Database Study**

**Reviewer #1, Comment #1**: The manuscript takes on an important but often overlooked issue: the drivers of price variation in radical bone tumor resections across the United States. The authors leverage a large national dataset and apply a thoughtful multivariate regression framework to explore hospital, payor, and state-policy factors shaping negotiated rates. From a policy perspective, this is a timely and highly relevant question, because the costs of oncologic surgery are escalating and transparency laws were designed precisely to illuminate such variability. I appreciate that the study highlights differences not only by hospital size and type but also by Medicaid expansion, Certificate of Need laws, and other state-level regulations. These are all important levers that could, in theory, guide cost containment strategies. The sheer scale of data (nearly 300,000 negotiated rates) is impressive and gives the study statistical power that most analyses of this type lack.

- **Author Response**: We thank Reviewer #1 for their positive feedback and for recognizing the importance of this topic and the strength of our dataset.

**Reviewer #1, Comment #2**: The most pressing issue is that the introduction jumps straight into orthopaedic oncology and cost transparency without giving readers a strong foundation in cancer biology and treatment strategies. Since this is ultimately a cancer surgery study, the narrative should begin with a broader perspective on the burden of cancer and the evolution of treatment approaches. For example, I recommend citing the review by D. Sonkin and A. Thomas, “Cancer Treatments: Past, Present, and Future” (2024), which was authored by the Chief of the US National Cancer Institute and provides a solid historical and clinical context for why access to surgery remains central even in the age of targeted therapies and immunotherapy. This would anchor the cost discussion within the larger story of cancer care.

- **Author Response**: This is an excellent point. We agree that providing a broader context on the role of surgery in modern cancer care will strengthen the manuscript's foundation. We have revised the introduction (**Page 4,** **Lines 58-61**) to include a discussion on the evolution of cancer treatments and the enduring importance of surgical resection, citing the suggested review.

**Reviewer #1, Comment #3**: In addition, the discussion could benefit from a deeper engagement with how financial variation intersects with equity and outcomes in cancer treatment. Right now, the paper notes that large hospitals and certain policy environments drive higher prices, but it does not really reflect on what this means for patients who require limb-sparing surgery for osteosarcoma or metastatic disease. It would strengthen the argument to cite and briefly discuss recent literature on cancer care costs globally.

- **Author Response**: Thank you for this insightful suggestion. We have expanded the discussion to better connect our findings on price variation to potential implications for patient equity and access to care (**Page 15, Lines 234-238**). We have also more specifically incorporated the literature around patient outcomes in the discussion (**Page 16-17, Lines 284-289**). and limitations. Additionally, we have expanded on our paragraph that contextualizes our findings within the global challenge of managing oncologic care costs by providing examples of difficulties with cost control in other countries (**Page 19, Lines 330-334**).

**Reviewer #1, Comment #4**: Similarly, the section on Medicaid reimbursement and access might draw on papers that show how reimbursement disparities can directly affect surgical oncology enrollment and availability. Without tying these policy findings back to patient outcomes, the paper risks reading like a pure health economics exercise rather than a piece of cancer policy research.

- **Author Response**: We agree that our discussion on Medicaid reimbursement was underdeveloped. We have now revised this section to explicitly discuss how lower reimbursement rates could potentially disincentivize provider participation and create access barriers for vulnerable patient populations, centering the discussion more around patient outcomes (**Page 16-17, Lines 284-289**). However, it must be noted that there are limited studies that specifically look at how rate variations for negotiated rates correspond with patient outcomes, as much of this literature is focused on reimbursement rates. Nonetheless, we have revised the discussion to incorporate this important point, as well as explicitly mentioned the lack of patient data in the limitations (**Page 20, Lines 362-363**).

**Reviewer #1, Comment #5**: Another point that needs attention is the interpretation of Certificate of Need and All-Payer Claims Database findings. The current manuscript highlights counterintuitive associations (e.g., CoN laws linked to higher rates), but the discussion does not fully engage with why these paradoxes might occur in the specific context of bone tumor surgery. One way to frame this is through the lens of unintended policy consequences, as transparency laws or market-entry restrictions may actually consolidate market power. Bringing in the broader oncology policy discussion, this would help place these observations in a larger framework of how reforms sometimes fail to achieve their intended cost-control effects.

- **Author Response**: Thank you for noting this oversimplification. As suggested by the reviewer, we now frame the counterintuitive results for CoN laws (**Page 17, Lines 296-300**) and APCDs (**Page 17, Lines 305-313**) through the lens of unintended policy consequences in the context of bone tumor surgery, discussing how these regulations might consolidate market power or lead to price leveling rather than cost reduction in a specialized market like orthopaedic oncology.

**Reviewer #1, Comment #6:** Lastly, the paper would benefit from more clarity on limitations. It is noted that reconstructive procedures are not captured, but this is a major caveat, because in real-world bone tumor surgery, reconstruction is often more resource-intensive than resection itself. That omission could substantially change the cost profile, and readers need a fuller acknowledgement of this. The writing also occasionally becomes too descriptive, listing results without embedding them into a coherent argument. I encourage the authors to rework the discussion so that each section not only reports the findings but also interprets them in light of cancer care delivery, patient equity, and future policy design.

- **Author Response**: We thank the reviewer for highlighting these important areas for improvement. We have rewritten the limitations section to give greater emphasis to the exclusion of reconstructive procedures, acknowledging this as a major factor that impacts the total cost profile (**Page 19, Lines 345-348**). The entire discussion section has also been revised to be more interpretive, guided by both reviewer comments.

**Reviewer #2, Comment #1**: This manuscript presents a timely and important analysis of price variation in radical bone tumor resections using a large national dataset derived from the Turquoise Health database. The authors investigate negotiated payor rates for CPT codes 24150 and 27365, examining associations with hospital characteristics, payor types, and state-level health policies. The study is well-structured, methodologically sound, and contributes to the literature on healthcare cost transparency and orthopaedic oncology.

**Reviewer #2, Comment #2:** Strengths 1. Novelty and Relevance: The study addresses a gap in the literature by focusing on specialized oncologic procedures, which are often excluded from broader cost analyses. The use of price transparency data for radical bone tumor resections is novel. 2. Robust Dataset: The analysis includes over 285,000 negotiated rates, providing strong statistical power and generalizability across U.S. hospitals. 3. Methodological Rigor: The use of multivariate linear regression and stratified analyses by CPT code is appropriate and well-executed. The authors control for multiple confounders and present confidence intervals and p-values clearly. 4. Policy Implications: The findings have implications for healthcare policy, particularly regarding the unintended consequences of Certificate of Need laws and price transparency mandates. 5. Clarity and Organization: The manuscript is well-written, with clear tables and logical flow from introduction to discussion.

- **Author Response**: Thank you for the positive feedback and for noting the strengths of this study. We appreciate the reviewers’ attention to detail and hope that the subsequent changes below help make this manuscript more rigorous for publication.

**Reviewer #2, Comment #3**: Suggestions/Concerns: 1. Scope of Procedure Pricing:
The analysis focuses solely on the resection portion of the procedure, excluding reconstructive components. This limitation is acknowledged but warrants further emphasis, as it may significantly affect total cost estimates and policy implications. Would the order of the CPT codes being submitted potentially influence the charges for the resection CPT specifically? I also think this limitation makes it challenging for a reader to understand the actual total costs/charges for the operation. If there is more or less variation in the reconstruction portions of the procedures, it could negate or widen the variation. Granted there are a lot of reconstructive options, but could a subanalysis be done for those who have a distal femur replacement? A proximal femur replacement? I’d also be curious what proportion of the procedures have a 22 Modifier submitted with them.

- **Author Response**: As noted in Reviewer #1 Comment #6, we have now expanded on this major limitation of our manuscript (**Page 19, Lines 345-348**) and how we are not capturing the full cost profile of patients undergoing bone tumor resection surgeries. While a sub-analysis of reconstruction codes is an excellent idea, the complexity and variability of these procedures (e.g., allograft, endoprosthesis, custom implants) make it difficult to perform a reliable analysis within the scope of the current study. However, we have noted this as a critical area for future research (**Page 19, Lines 350-352**). Additionally, to our knowledge, the order of CPT codes should not affect the billing of the procedure. Given that this is a study of negotiated payor rates, and not reimbursement rates, we are unable to comment on how prices change based on the order of the procedure.

**Reviewer #2, Comment #4**: 2. Data Source Limitations: While the Turquoise Health database is a valuable resource, the potential for incomplete or inconsistent reporting by hospitals should be discussed more thoroughly, especially regarding the reliability of negotiated rates. It would be great if possible to only include those hospitals who actually billed at least one of those procedures. I’d be curious how many of these resections are happening at particularly small hospitals, or if the costs at hospitals who actually perform these operations regularly are higher or lower than those that do not do these procedures. Given this are largely tertiary care type procedures, there may be many hospitals where neither of these CPTs is utilized in a given year, making the negotiated rate rather unimportant. Those hospitals with under 100 beds may not do these procedures, making that hospital less keen to negotiate for a higher rate. I think this limitation needs to be made more clear in the limitations section.

- **Author Response**: This is an important point that we had not sufficiently addressed. We have added two sentences to our limitations section expanding on this limitation of the Turquoise Health database (**Page 19-20, Lines 355-359**).

**Reviewer #2, Comment #5**: Is it possible to evaluate by region using this database? It would be interesting to know whether certain US regions (by US Census subgroups, for example) are significantly different.

- **Author Response**: We thank the reviewer for this interesting suggestion. We have now added a sub-analysis of changes in rates according to the 9 U.S. Census Bureau divisions. We have updated Table 1 to descriptively list the number of rates in each region, as well as added “Region” into both multivariable regressions (**Table 2 and Table 4**). Given that the inclusion of another variable shifts all results, we have now updated the correct rates in the new adjusted analysis for both CPT codes throughout the manuscript. We have added a discussion (**Page XXX, Lines 259-268**). of these findings into the results and appropriately updated all in-text values in the results.

**Reviewer #2, Comment #6**: 3. Discussion: The discussion mentions that for patients with primary or metastatic disease the lack of price predictability may create uncertainty. For nearly all patients in this condition, the cost of their operation is but a small part of the price of their overall care. I do not think this conclusion is justified by the data presented. You’d have to show that the cost of the resection (and reconstruction) constitute a major part of the total cost of care. I think this sentence should be omitted.

- **Author Response**: We thank the reviewer for noting this issue. We have removed this sentence from the discussion (**Page 14, Lines 234-236**).

**Reviewer #2, Comment #7**: I think the paragraph about higher cost at larger hospitals should reflect that there is no direct data here that I saw suggesting that these procedures actually happen at smaller hospitals. It would be really valuable to have some data to that effect. While this is a potential issue, it may be a small or a non-issue if few of these operations are actually happening at small hospitals. Similarly, it is hard to place context for the findings on critical access hospitals, as we are not at all sure how often these operations happen at critical access hospital. At least in my area, I think any of these patients who might benefit from one of these operations would be transferred to another hospital.

- **Author Response**: This is an excellent point that ties back to Comment #4. We have revised our discussion of hospital size and type (including CAHs) to be more cautious in our interpretation, acknowledging that negotiated rates from smaller facilities may not reflect actual case volumes (**Page 19-20, Lines 355-359**).

**Reviewer #2, Comment #8**: With suggestions incorporated as above, I think this paper could highlight that the negotiated rates are highly variable. Recognizing that these are negotiated rates, which do not necessarily reflect where/if these procedures are happening, would be an improvement. It would also be useful to understand if this degree of variability is similar to or different to the variability for more common procedures. As in: is this a problem throughout US healthcare, or is somehow the problem more of an issue for humerus/femur bone resections.

- **Author Response**: We have now emphasized one of the main conclusions of the study to be regarding the high-rate variations as examined through the variables included in this study (**Page 21, Lines 371-372**). We have also contextualized our findings within other articles discussing rate variations (**Page 15, Lines 234-238**).

**Reviewer #3 Comments:** On review of this reviewers’ comment, we felt that it was likely meant for another paper. For example, the reviewer makes comments about figures pertaining to signaling pathways, resistance mechanisms for chemotherapy/immunotherapy, and discussions about tumor biology, which are not relevant to this manuscript’s content. Thus, we have not responded to Reviewer #3 concerns, but remain available for additional changes as requested.
