# Supplementary material for: Exploring the drivers of price variation in orthopaedic radical bone tumor resection: A nationwide database study
Source: PLoS One. 2026 Feb 26;21(2):e0343676. doi: 10.1371/journal.pone.0343676 (PMC12944713; doi:10.1371/journal.pone.0343676)
Supplement: Attachment — Submitted filename: Revised radresection manuscript v4.docx [file pone.0343676.s007.docx]

**Exploring the Drivers of Price Variation in Radical Bone Tumor Resection: A Nationwide Database Study**

Devika A. Shenoy^1^, BS; William C. Cruz^2^, BS; Shamik Bhat^3,4^, BS; Katelyn Parsons^1^, BS; Aaron D. Therien^1^, MS; Kevin A. Wu^5^, MD; Christian A. Pean^1^, MD, MS; William C. Eward^1^, DVM, MD

^1^Department of Orthopaedic Surgery, Duke University School of Medicine, Durham, NC, USA

^2^School of Osteopathic Medicine, Campbell University, Durham, NC, USA

^3^School of Public Policy, Princeton University, Princeton, NJ, USA

^4^School of Medicine, Yale University, New Haven, CT, USA

^5^Department of Orthopedic Surgery, Icahn School of Medicine at Mount Sinai, New York City, NY, USA

Corresponding Author:

Shamik Bhat, BS

shamik.bhat@yale.edu

Tables: 5

Manuscript Word Count: 3047

Abstract Word Count: 237

Conflicts of Interest/Funding: None relevant to this study.

Data Availability Statement: The primary data used by this study was exported under a licensing agreement with a third party, Turquoise Health.

Keywords: Orthopaedic Surgery; Negotiated Payor Rates; Health Policy; Price Transparency; Cancer; Metastatic Bone Disease; Bone Cancer

**Abstract**

**Background:** Radical resection of bone tumors is a clinically effective but costly procedure. Despite the implementation of federal price transparency mandates, little is known about the nationwide variation in negotiated prices for these specialized oncologic surgeries. This study aimed to quantify the variation in negotiated rates for radical resection of the humerus and femur/knee and identify associated hospital, payor, and state-policy drivers.

**Methods:** This cross-sectional study analyzed hospital-negotiated payor rates from the Turquoise Health database for current procedural terminology (CPT) codes 24150 (humerus resection) and 27365 (femur/knee resection). Multivariate linear regression was used to determine the associations between of hospital size and type, payor class, and state-level policies (Medicaid expansion, Certificate of Need [CoN] laws, All-Payer Claims Database [APCD] mandates, and NP scope of practice) on negotiated payor rates.

**Results:** A total of 285,857 negotiated rates were analyzed. Significant price variation was observed across all factors. Large hospitals (>1000 beds) and Critical Access Hospitals (for femur/knee resection only) commanded significantly higher rates. CoN laws were associated with higher prices for both procedures (+$374.59 and +$641.59, respectively), as were APCD mandates for femur/knee resections (+$579.76). Medicare Advantage plans paid inconsistently compared to commercial plans, paying more for humerus but substantially less for femur/knee resections.

**Discussion:** Negotiated prices for radical bone tumor resection are highly variable and influenced by a complex interplay of market dynamics, challenging the assumption that price transparency alone can standardize healthcare costs for specialized care.

**Introduction**

The management of primary and metastatic bone tumors has evolved greatly over the past few decades, with new options for advanced surgical reconstructions and targeted immunotherapy providing patients with multifaceted treatment options.[1-4] While primary bone cancers account for only 0.2% of primary malignancies in the U.S., approximately 5.1% of patients with other malignancies report developing bone metastases, with the national cost burden for patients with metastatic bone disease estimated at $12.6 billion dollars in 2007.[5-7] Despite advancements in medical therapies, surgical management remains at the cornerstone of ensuring local tumor control, and preserving access to high-quality surgical care is essential for patients.[8] For example, radical resection of bone tumors involves removing sections from long bones, often in the setting of primary bone tumors such as osteosarcoma, or for metastatic bone disease.[9-11] While clinically effective in providing local tumor control and preserving limb function[9-11], there is limited knowledge regarding the costs of these procedures in the past decade.

Due to their impacts on both direct patient costs and costs to the healthcare system, the financial dynamics of major surgical procedures are increasingly under scrutiny.[12-17] The recent implementation of the Centers for Medicare & Medicaid Services (CMS) Price Transparency Rule provides an opportunity to address these concerns.[18] By mandating that hospitals publicly disclose their privately negotiated rates with insurers, this policy has offered the ability to investigate the true, market-driven prices for specific procedures on a national scale, making it possible to explore the financial landscape of specialized oncologic surgeries. Recent studies leveraging price transparency data have uncovered significant, often unexplained, variation in the negotiated prices for common procedures in both general orthopaedics and surgical oncology.[13, 14, 16, 17, 19] For example, one cross-sectional study of 15,013 Medicare beneficiaries found large, regional variations in negotiated payor rates for common oncologic operations, as well as mild associations between high rates and adverse clinical outcomes.[17] Additionally, another study found that healthcare policies, such as Medicaid Expansion and Certificate of Need (CoN) status, were associated in large variations in payor rates for total hip arthroplasty.[13]

However, it is unknown if similar price variability exists for high-stakes, less common procedures like the radical resection of bone tumors. Therefore, the present study leverages a national price transparency database to analyze two analogous but distinct procedures, radical resection of a tumor in the humerus and the femur or knee, the most common locations for such procedures. The objectives of this study were to aim to quantify the nationwide variation in their negotiated prices along common hospital, payor, and state-policy level factors. While the specific policies analyzed are unique to the U.S., the findings on how market dynamics influence the valuation of complex surgical care have global implications for health systems navigating the challenges of cost containment and price transparency.

**Methods**

*Overview*

This is a cross-sectional study evaluating negotiated prices between hospitals and payors from the Turquoise Health (TQH) “Clear Rates” Database, which aggregates U.S. hospital-reported negotiated payor rates for common procedures by current procedural terminology (CPT) code.[20]

*Inclusion Criteria*

CPT codes evaluated by this study included 24150 (i.e. radical resection of tumor, shaft of humerus or distal humerus) and 27365 (i.e. radical resection of tumor, femur or knee). Data were analyzed in one overall cohort containing both CPT codes, and then individually within a “radical resection of humerus” and a “radical resection of femur/knee” cohort.

For this analysis, we only included rates from the TQH database that had complete payor and regional information. Following studies with similar methodologies, we then excluded outliers within the top and bottom 10% of all negotiated rates.

*Data Source*

Data was extracted from TQH on December 31^st^, 2024, under a licensing agreement, and included procedure rate, the type of payor, total bed range of the hospital (i.e. a proxy for hospital size), hospital type, and hospital location. Data on Medicaid expansion status and CoN regulations were extracted from the Kaiser Family Foundation and the National Conference of State Legislatures using 2024 data, and was extracted on December 31^st^, 2024.[21, 22] CON laws are intended to limit healthcare cost growth by restricting market entry and expansion; their association with negotiated prices in orthopaedics is a key area of continued research.[13-15] Scope of practice regulations for advanced practice providers were included as an additional proxy for market competition, which can influence overall procedural rates. For example, broader practice authority may expand access to conservative or postoperative care options, potentially impacting surgical case volumes or procedure demands.[23] Data on NP independent practice regulations were extracted from the 2024 American Association of Nurse Practitioners data on July 1^st^, 2025.[24] Lastly, state-mandated All-Payer Claims Database (APCD), which mandates the reporting of all public and private health claims, are a direct price transparency mechanism that may have an impact on negotiation.[25] Data on state participation in All-Payer Claims Database (APCD) initiatives were extracted from the University of New Hampshire (UNH) and the National Association of Health Data Organizations (NAHDO) database on July 1^st^, 2025.[25]

*Hospital and Payor-Level Variables*

Procedure rates are standardized in 2024 U.S. Dollars. Categories for the type of payor included commercial, managed Medicaid, Medicare Advantage, dual Medicare-Medicaid, exchange, Veteran Affairs (VA), and worker’s compensation. Total bed range was defined as a categorical variable with 6 options, as reported by TQH: 1-100, 100-300, 300-500, 500-1000, 1000-5000, 1500+. Hospital type was reported by TQH. Critical Access Hospitals (CAHs) are hospitals designated by CMS to provide care within a geographic area without other hospitals in a 35-mile radius.[26] All other hospitals were designated as acute care hospitals.

*State Policy Variables*

Medicaid expansion status, CoN regulations, and state-mandated APCD participation were all defined as binary variables (yes/no). NP scope-of-practice regulations were categorized into “full practice,” “restricted practice,” and “no practice.”

*Regional Variables*

Nine regions were defined according to the U.S. Census Bureau using the state of the listed hospital: New England, Mid-Atlantic, East North Central, West North Central, South Atlantic, East South Central, West South Central, Mountain, and Pacific.[27] A full breakdown of states in each listed region is provided in the Appendix (**Table S1).**

*Statistical Analysis*

First, a descriptive analysis was conducted to summarize cohort characteristics using frequencies, percentages, means (SD), and medians (IQR). Second, a multivariate linear regression was performed to determine the influence of hospital, payor, regional, and state policy factors on negotiated rates. All regressions control for every included variable in the study. Regression coefficients represent the mean difference in U.S. dollars between the evaluated variable and the reference group. A p-value ≤ 0.05 was considered statistically significant for all analyses, which were conducted in R (Version 4.4.1).

*Ethics Approval*

This study includes publicly available data and negotiated payor rates from the Turquoise Health Database. Given that negotiated payor rates are general listed prices publicly posted by a hospital, these are not associated with patients or hospital encounters. Thus, this study was approved as exempt by our Institutional Review Board (#Pro00112768).

**Results**

*Cohort Details*

A total of 285,857 negotiated payor rates were included for analysis (**Table 1).** This comprised 177,728 rates for radical resection of the humerus (CPT 24150) and 108,129 rates for radical resection of the femur/knee (CPT 27365). For the humerus cohort, the median payor rate was $6,308.38 (IQR: $2,982.82 – $7,218.64), and the mean rate was $5,645.95 (SD: $2,626.63). For the femur/knee cohort, the median rate was $4,388.24 (IQR: $2,533.43 – $7,916.16), and the mean rate was $5,575.91 (SD: $3,613.73). The highest percentage of rates came from the Middle Atlantic geographic region (40,233 (22.6%) for 24150; 29,566 (27.3%) for 27365).

**Table 1: Overview of Included Negotiated Payor Rates for Radical Resection of Bone Tumors**

| **Variable** | **CPT 24150^a^** | **CPT 27365^b^** |
| --- | --- | --- |
| Number of Payor Rates | 177,728 | 108,129 |
| Median Payor Rate (IQR) | $6,308.38 ($2,982.82 – $7,218.64) | $4,388.24 ($2,533.43 – $7,916.16) |
| Mean Payor Rate (SD) | $5,645.95 ($2,626.63) | $5,575.91 ($3,613.73) |
| *Total Bed Range of Hospital (N (%))* | | |
| 1 - 100 | 51,183 (28.8%) | 32,753 (30.3%) |
| 100 - 300 | 64,454 (36.3%) | 36,928 (34.2%) |
| 1000 - 1500 | 1,731 (1.0%) | 1,095 (1.0%) |
| 300 - 500 | 35,477 (20.0%) | 23,151 (21.4%) |
| 500 - 1000 | 22,148 (12.5%) | 11,634 (10.8%) |
| 1500 + | 2,735 (1.5%) | 2,568 (2.4%) |
| *Payor Class (N (%))* | | |
| Commercial | 100,590 (56.6%) | 79,224 (73.3%) |
| Dual | 1,369 (0.8%) | 259 (0.2%) |
| Managed Medicaid | 17,264 (29.6%) | 13,428 (12.4%) |
| Medicare Advantage | 52,635 (29.6%) | 13,172 (12.2%) |
| Veterans Affairs | 3,126 (1.8%) | 800 (0.7%) |
| Workers' Compensation | 2,744 (1.5%) | 1,246 (1.2%) |
| *Hospital Type (N (%))* | | |
| Acute Care | 166,686 (93.8%) | 94,539 (87.4%) |
| Critical Access | 11,042 (6.2%) | 13,590 (12.6%) |
| *U.S. Census Bureau Division* | | |
| Middle Atlantic | 40,233 (22.6%) | 29,566 (27.3%) |
| New England | 11,617 (6.5%) | 5,225 (4.8%) |
| East North Central | 30,973 (17.4%) | 9,355 (8.6%) |
| East South Central | 15,367 (8.6%) | 8,060 (7.5%) |
| Mountain | 6,060 (3.4%) | 9,753 (9.0%) |
| Pacific | 17,818 (10.0%) | 9,221 (8.5%) |
| South Atlantic | 30,906 (17.4%) | 14,729 (13.6%) |
| West North Central | 11,812 (6.6%) | 12,654 (11.7%) |
| West South Central | 12,942 (7.3%) | 9,566 (8.8%) |
| Abbreviations: IQR = Interquartile Range; SD = Standard Deviation; CPT = Current Procedural Terminology Code  ^a^CPT 24150 = Radical resection of tumor, shaft of humerus or distal humerus  ^b^CPT 27365= Radical resection of tumor, femur or knee | | |

Most rates for both procedures came from hospitals with 100-300 beds (36.3% for humerus, 34.2% for femur/knee). Commercial payors represented the largest payor class for both cohorts, accounting for 56.6% of humerus resection rates and 73.3% of femur/knee resection rates. Rates from acute care hospitals were most common, representing 93.8% of the humerus cohort and 87.4% of the femur/knee cohort. A full overview is available in Table 1.

*Radical Resection of Humerus Cohort*

After controlling for other variables, a multivariate linear regression for the humerus resection cohort (CPT 24150) revealed several significant factors associated with payor rates (**Table 2).** Compared to hospitals with 0-100 beds, rates were significantly higher in facilities with 100-300 beds (+$163.47, p<0.001), 1000-1500 beds (+$469.45, p<0.001), and 1500+ beds (+$797.46, p<0.001). Rates were lower in hospitals with 500-1000 beds (-$98.48, p<0.001). Relative to Commercial payors, rates were higher Workers' Compensation (+$1,543.66, p<0.001) and Medicare advantage ($71.18, p<0.001) plans. Rates for Managed Medicaid (-$2,016.69, p<0.001) and Veterans Affairs (-$295.97, p<0.001) were lower compared to Commercial payors. CAHs had rates that were $703.76 lower than acute care hospitals (p<0.001). In the regional analysis, with the exception of the East North Central region, all regions had higher rates in comparison to the Middle Atlantic region varying between $172.04 higher (p<0.001, East South Central) and $1,769.43 higher (p<0.001, New England).

**Table 2: Multivariable Linear Regression for Payor Rates within the Radical Resection of Humerus Cohort**

| Variable | Estimate (USD)^a^ | p-value | Lower Limit, 95% Confidence Interval (USD) | Upper Limit, 95% Confidence Interval (USD) |
| --- | --- | --- | --- | --- |
| *Total Bed Range of Hospital* | | | | |
| 1 - 100 | Reference | Reference | Reference | Reference |
| 100 - 300 | $163.47 | <0.001* | $130.68 | $196.25 |
| 300 - 500 | $-34.95 | 0.0685 | $-72.54 | $2.65 |
| 500 - 1000 | $-98.48 | <0.001* | $-140.38 | $-56.58 |
| 1000 - 1500 | $469.45 | <0.001* | $348.82 | $590.08 |
| 1500 + | $797.46 | <0.001* | $585.88 | $1009.03 |
| *Payor Class* | | | | |
| Commercial | Reference | Reference | Reference | Reference |
| Dual | $-14.97 | 0.83 | $-155.26 | $125.31 |
| Managed Medicaid | $-2016.69 | <0.001* | $-2060.35 | $-1973.03 |
| Medicare Advantage | $71.18 | <0.001* | $39.68 | $102.68 |
| Veterans Affairs | $-295.97 | <0.001* | $-389.10 | $-202.84 |
| Workers' Compensation | $1543.66 | <0.001* | $1443.41 | $1643.90 |
| *Hospital Type* | | | | |
| Acute Care | Reference | Reference | Reference | Reference |
| Critical Access | $-703.76 | <0.001* | $-764.73 | $-642.79 |
| *U.S. Census Bureau Division* | | | | |
| Middle Atlantic | Reference | Reference | Reference | Reference |
| New England | $1769.43 | <0.001* | $1563.83 | $1975.03 |
| East North Central | $-983.21 | <0.001* | $-1059.54 | $-906.88 |
| East South Central | $172.04 | 0.002* | $63.93 | $280.15 |
| Mountain | $197.18 | <0.001* | $117.13 | $277.23 |
| Pacific | $1576.57 | <0.001* | $1485.99 | $1667.15 |
| South Atlantic | $1143.36 | <0.001* | $1041.10 | $1245.63 |
| West North Central | $625.83 | <0.001* | $545.56 | $706.10 |
| West South Central | $313.16 | <0.001* | $236.22 | $390.10 |
| Abbreviations: USD = United States Dollars  ^a^ A “-” symbol preceding the estimate corresponds to a reduction in payor rates in comparison to the reference group.  *Statistically significant, p < 0.05 | | | | |

A sub-analysis of health policies revealed several significant associations with payor rates for the humerus resection cohort **(Table 3).** States with Medicaid expansion had rates that were $274.64 higher than states without it (p<0.001), states with CON laws were associated with rates that were $348.25 higher than states without such laws (p<0.001), and the existence of an APCD mandate was not significantly associated with a change in rates (p=0.97). Compared to states with no independent practice for nurse practitioners, rates were higher in states with restricted practice ($662.08, p<0.001) and lower in states with full practice (-$226.29, p<0.001).

**Table 3: Multivariable Linear Regression for Payor Rates within the Radical Resection of Humerus Cohort, Health Policy Sub-Analysis**

| Health Policy Variable^a^ | Estimate (USD)^b^ | p-value | Lower Limit, 95% Confidence Interval (USD) | Upper Limit, 95% Confidence Interval (USD) |
| --- | --- | --- | --- | --- |
| Medicaid Expansion Status | | | | |
| No | Reference | Reference | Reference | Reference |
| Yes | $274.64 | <0.001* | $214.64 | $334.64 |
| Certificate of Need Status | | | | |
| No | Reference | Reference | Reference | Reference |
| Yes | $348.25 | <0.001* | $288.25 | $408.26 |
| Scope of Independent Practice Regulations for Nurse Practitioners | | | | |
| No Practice | Reference | Reference | Reference | Reference |
| Restricted Practice | $662.08 | <0.001* | $604.24 | $719.92 |
| Full Practice | $-226.29 | <0.001* | $-305.22 | $-147.35 |
| All Payor-Claims (i.e. Price Transparency) Database Mandate | | | | |
| No | Reference | Reference | Reference | Reference |
| Yes | $0.78 | 0.97 | $-34.24 | $35.80 |
| Abbreviations: USD = United States Dollars  ^a^These are state-level variables and denote whether the negotiated payor rate was found within a state that implemented each respective policy.  ^b^A “-” symbol preceding the estimate corresponds to a reduction in payor rates in comparison to the reference group.  *Statistically significant, p < 0.05 | | | | |

*Radical Resection of Femur/Knee Cohort*

For the femur/knee resection cohort (CPT 27365), a multivariate linear regression identified several significant variables impacting payor rates (**Table 4**). Compared with hospitals with 0-100 beds, rates were significantly higher in hospitals with 1000-1500 beds (+$1,753.12, p<0.001), and 1500+ beds (+$3,409.96, p<0.001). Rates were lower in all other facility sizes. In the regional analysis, all regions had rates that were higher than the Middle Atlantic division, ranging from +$252.58 (p<0.001, West South Central) to $3,791.18 (p<0.001, South Atlantic).

**Table 4: Multivariable Linear Regression Results for Payor Rates within the Radical Resection of Femur/Knee Cohort**

| Variable | Estimate (USD)^a^ | p-value | Lower Limit, 95% Confidence Interval (USD) | Upper Limit, 95% Confidence Interval (USD) |
| --- | --- | --- | --- | --- |
| *Total Bed Range of Hospital* | | | | |
| 1 - 100 | Reference | Reference | Reference | Reference |
| 100 - 300 | $-236.27 | <0.001* | $-292.93 | $-179.60 |
| 300 - 500 | $-441.46 | <0.001* | $-502.86 | $-380.07 |
| 500 - 1000 | $-296.51 | <0.001* | $-368.39 | $-224.62 |
| 1000 - 1500 | $1753.12 | <0.001* | $1561.12 | $1945.11 |
| 1500 + | $3409.96 | <0.001* | $3165.47 | $3654.45 |
| *Payor Class* | | | | |
| Commercial | Reference | Reference | Reference | Reference |
| Dual | $-2218.99 | <0.001* | $-2611.39 | $-1826.60 |
| Managed Medicaid | $-2082.38 | <0.001* | $-2142.15 | $-2022.61 |
| Medicare Advantage | $-2946.11 | <0.001* | $-3007.68 | $-2884.53 |
| Veterans Affairs | $-2156.83 | <0.001* | $-2387.21 | $-1926.45 |
| Workers' Compensation | $-375.72 | <0.001* | $-557.31 | $-194.13 |
| *Hospital Type* | | | | |
| Acute Care | Reference | Reference | Reference | Reference |
| Critical Access | -$1,073.85 | <0.001* | -$1149.93 | -$997.76 |
| *U.S. Census Bureau Division* | | | | |
| Middle Atlantic | Reference | Reference | Reference | Reference |
| New England | $2204.43 | <0.001* | $2010.22 | $2398.63 |
| East North Central | $2264.97 | <0.001* | $2139.26 | $2390.68 |
| East South Central | $2730.81 | <0.001* | $2557.84 | $2903.79 |
| Mountain | $2203.69 | <0.001* | $2098.30 | $2309.08 |
| Pacific | $271.28 | <0.001* | $171.36 | $371.21 |
| South Atlantic | $3791.18 | <0.001* | $3638.89 | $3943.48 |
| West North Central | $3143.80 | <0.001* | $3050.50 | $3237.09 |
| West South Central | $252.58 | <0.001* | $129.03 | $376.13 |
| Abbreviations: USD = United States Dollars  ^a^ A “-” symbol preceding the estimate corresponds to a reduction in payor rates in comparison to the reference group.  *Statistically significant, p < 0.05 | | | | |

The health policy sub-analysis for the femur/knee cohort also found multiple significant associations with payor rates **(Table 5).** In states with Medicaid expansion, rates were $1399.41 higher than in non-expansion states (p<0.001) and states with CON laws had rates that were $667.98 higher than states without such laws (p<0.001). Relative to states with no independent practice for nurse practitioners, rates were significantly higher in states with restricted practice (+$1563.49, p<0.001) and lower in states with full practice (-$250.79, p<0.001). The presence of an APCD mandate was associated with rates that were $1,231.24 higher in comparison to states without it (p<0.001).

**Table 5: Multivariable Linear Regression Results for Radical Resection of Femur/Knee Cohort, Health Policy Sub-Analysis**

| Health Policy Variable^a^ | Estimate (USD)^b^ | p-value | Lower Limit, 95% Confidence Interval (USD) | Upper Limit, 95% Confidence Interval (USD) |
| --- | --- | --- | --- | --- |
| Medicaid Expansion Status | | | | |
| No | Reference | Reference | Reference | Reference |
| Yes | $1399.41 | <0.001* | $1310.93 | $1487.89 |
| Certificate of Need Status | | | | |
| No | Reference | Reference | Reference | Reference |
| Yes | $667.98 | <0.001* | $578.80 | $757.15 |
| Scope of Independent Practice Regulations for Nurse Practitioners | | | | |
| No Practice | Reference | Reference | Reference | Reference |
| Restricted Practice | $1563.49 | <0.001* | $1477.68 | $1649.30 |
| Full Practice | $-250.79 | <0.001* | $-362.50 | $-139.07 |
| All Payor-Claims (i.e. Price Transparency) Database Mandate | | | | |
| No | Reference | Reference | Reference | Reference |
| Yes | $1231.24 | <0.001* | $1174.38 | $1288.09 |
| Abbreviations: USD = United States Dollars  ^a^These are state-level variables and denote whether the negotiated payor rate was found within a state that implemented each respective policy.  ^b^A “-” symbol preceding the estimate corresponds to a reduction in payor rates in comparison to the reference group.  *Statistically significant, p < 0.05 | | | | |

**Discussion**

To our knowledge, this is the first study to leverage a national price transparency database to investigate the negotiated payor rates for radical resection of bone tumors. Our analysis of over 285,000 negotiated rates for radical resection procedures of the humerus and femur/knee reveals significant price variation driven by hospital, payor, regional, and state-level policy factors. These findings are similar to previous studies that identify large, often unexplained variations in negotiated payor rates.[13, 14, 16, 17] For patients with primary or metastatic bone disease, who often require complex and costly care, this variability raises important questions about health equity in a specialized field where patient choice may be limited by geography and clinical expertise.

A primary finding of this study is that hospital size is a significant driver of negotiated rates. For both procedures, the largest hospitals (1000+ beds) had substantially higher prices than smaller facilities. This is particularly pronounced for femur/knee resections, where rates at the very largest hospitals were approximately $3400 higher than at the smallest. This may reflect the market power of large hospitals, often with academic sarcoma centers that can handle complex cases and consequently may have greater leverage in negotiations with payors.[28, 29] Given that most orthopaedic oncologists who perform these procedures routinely are located in academic medical centers, these findings may mean patients and health systems have no choice but to pay for the higher-cost procedures, effectively creating a price-inelastic market where transparency alone is unlikely to foster competition or reduce costs. Additionally, although prior studies have found that larger hospitals have higher prices than smaller hospitals[28, 29], we observed a non-linear trend in the present study, with mid-sized hospitals (300-1000 beds) having inconsistently high or low rates compared to both the smallest and largest facilities. This may suggest a competitive disadvantage for these institutions, which may lack the negotiating power of large systems (e.g., those with academic sarcoma centers). However, when considering hospital type, CAHs had significantly lower rates for both procedures. The lower rate for the humerus procedure at CAHs may align with expectations of lower-case volume or complexity in rural settings.[30] Together, these findings suggest that market power, whether in the context of specialized expertise of large academic centers or the geographic monopoly of small rural CAHs, may be a driver of high prices for specialized orthopaedic oncology care.

Our analysis also uncovered regional differences in negotiated rates that persist even after controlling for hospital, payor, and state-policy factors. Using the Middle Atlantic (i.e., NY, NJ, PA) as the reference, nearly all other U.S. regions had significantly higher prices for both procedures. The variation was particularly pronounced for femur/knee resections, where rates in the South Atlantic (e.g., NC, SC, VA) were nearly $3,800 higher, and rates in the West North Central region (e.g., ND, SD, MN) were over $3,100 higher. Notably, the East North Central region was a significant outlier, with humerus resection rates nearly $1,000 lower than in the Middle Atlantic. Such large geographic variations may reflect underlying differences in regional market concentration, the negotiating leverage of dominant hospital systems in certain areas, or cost-of-living differences not fully captured by other variables.

The influence of payor type also revealed significant variability. Medicaid plans were associated with the lowest payor rates for both procedures. This finding is consistent with prior literature in orthopaedics, and may reflect limits on Medicaid reimbursement.[31, 32] While lower rates are likely beneficial to patients with reduced cost-sharing, it is also important to ensure lower prices do not disincentivize providers from offering the service. For example, in neurosurgical populations, lower reimbursement rates for common procedures were associated with lower provider enrollment.[33] Future studies should evaluate access to orthopaedic oncology care in populations with Medicaid insurance to ensure that such patients are not facing additional barriers out of the scope of this study. Other insurance subtypes also showed unclear variability. For humerus resections, Medicare Advantage plans had higher rates than commercial plans (+$71), while for femur/knee resections, they paid significantly less (-$2,946). These differences suggest that the balance of power between a given hospital and a given Medicare Advantage plan can lead to vastly different prices for similar services. Taken together, these wide variations by insurer reveal a variable pricing system where the cost of essential cancer surgery is determined not only by clinical value, but also by the specific negotiating power and policies of a patient's insurance plan. However, whether this variability translates to differences in patient outcomes is a critical, yet unknown, question in orthopaedic oncology. In one gastrointestinal surgical cancer population, Sankaran et al found that despite high rate variation between similar providers, the median payor rate was not associated with mortality or major postoperative complications.[17] Future studies are needed to understand the relationship between payor-driven variation in procedure costs, access to care, and outcomes for orthopaedic oncology patients.

At the state policy level, our findings were often counterintuitive. The presence of CoN laws, which are intended to control costs by limiting market entry and ensuring a healthcare “need”, were associated with significantly higher prices for both procedures. This finding conflicts with other studies in orthopaedic surgery, that found associations between CoN laws and lower prices.[12, 14, 15] In the context of orthopaedic oncology care, which already may face higher prices due to hospital-level factors, it is possible that CON laws may inadvertently grant preexisting hospitals greater market power, allowing them to negotiate higher rates. Because the market for radical bone tumor resection is already limited to a small number of tertiary care centers with the requisite multidisciplinary expertise and infrastructure, CoN laws may effectively shield these centers from competition. This may further increase prices and limit access to care for patients who need radical resections.

Medicaid expansion was linked to higher negotiated rates, consistent with prior research.[12, 14, 15] However, state-mandated APCDs, a direct price transparency mechanism, were not associated with humerus resection payor rates but were associated with $1,231 higher payor rates for femur/knee resections. In the case of radical resection of bone tumors, this contradicts the logic that transparency should foster competition and lower prices. This paradox may be specific to high-acuity, low-volume procedures where patient choice is driven by clinical expertise rather than cost. For example, a patient with a bone sarcoma may decide where to seek care based on the reputation of the surgical team and the geographically feasiblity, rather than by comparing listed prices.[34] In this price-inelastic market, transparency may not empower patients to "shop" for lower prices, but instead may allow hospitals to adjust their own rates upward toward those of their high-cost peers knowing that patients will be restricted to their institutions, leading to an inflationary effect. Thus, in the radical bone tumor resection market, it is possible that transparency may allow lower-priced providers to increase prices to the market price, which may be inflated at baseline by the state- and hospital-level factors mentioned above.

The findings for NP scope of practice laws have implications for understanding the role of advanced practice providers in specialized surgical markets. For humerus resections, broader NP practice authority was linked to lower surgical rates, supporting the theory that an expanded workforce could increase competition or access to non-operative care.[23] Yet, for femur/knee resections, the opposite was true, with broader NP practice linked to significantly higher rates. This discrepancy may indicate that the role of advanced practice providers differs by procedure complexity and should be explored in future research. It is possible that these differences reflect differences in NP role based on tumor location. In the lower extremity, resections are often more likely to be essential due to lower extremity weight bearing requirements. For patients with upper extremity tumors, is possible that access to NPs can result in alternatives to surgery, such as injections and counseling about weight bearing limitations. On the contrary, the same access to NPs for patients with lower extremity lesions could result in more rapid facilitation of a surgical intervention.

While this analysis is specific to the multi-payer healthcare system of the U.S., health systems worldwide, whether single-payer or market-based, are grappling with the challenge of controlling costs for specialized oncologic care, while ensuring access and quality.[35-37] For example, even in universal healthcare systems (e.g. Canada) where the government is permitted to negotiate the price of cancer treatments with manufacturers and direct patient-facing medical costs are lower, patients still experience high levels of financial distress related to their cancer care.[38, 39] Our findings serve as a case study for variable market dynamics, demonstrating that well-intentioned policies like market entry regulation (CON laws) and price transparency mandates can have counterintuitive, and even inflationary, effects on prices. For international policymakers considering market-based reforms or greater price disclosure, this study highlights that simply revealing prices or limiting provider supply does not guarantee cost containment. Further efforts are needed to determine the best way to limit costs. The complex interplay between provider access, regulatory frameworks, and negotiating power can subvert policy goals, which should be kept in mind when establishing fair and predictable pricing for complex medical procedures.

This study has several limitations. Most importantly, the prices included in this study account for the resection portion of the procedure, but they do not account for any reconstructive portion(s). In clinical practice, reconstruction is an integral and often more resource-intensive component of limb-sparing surgery than the resection itself. The reconstructive portion may also have its own significant price variability associated with it due to market forces that drive implant prices. Consequently, the price variability observed in our study may not be representative of the complete episode of care associated with the surgical treatment of bone tumors. Future studies should incorporate all elements of bone tumor resection surgery to develop a complete cost profile for patients undergoing these procedures, including grafts, implants, and custom endoprostheses. Second, data were extracted from TQH, relying on the accuracy of each institution’s disclosed negotiated rates. Hospitals or insurers may report only partial or grouped costs, potentially underestimating or overestimating total procedure expenses. Similarly, an additional limitation of this data source is that it includes rates from all hospitals, irrespective of whether they actually perform these complex procedures. Many smaller hospitals may list a negotiated rate for these CPTs but never perform the operation. This is particularly relevant for the interpretation of findings related to small hospitals and Critical Access Hospitals, whose negotiated rates may be theoretical rather than based on actual clinical volume. Third, this analysis only considers negotiated rates and does not account for variations in true reimbursement rates or direct out-of-pocket costs incurred by patients. Fourth, this study did not incorporate clinical outcomes (e.g., surgical complication rates) or patient-reported outcomes (e.g., financial toxicity or quality-of-life measures), which would be necessary to gauge the true value and cost-efficacy of each intervention in the long-term. Finally, our analysis was limited to two CPT codes for radical resection of long bones, and the findings may not be generalizable to other oncologic procedures or anatomical locations. While this approach was chosen to allow for a direct evaluation of two common procedures, future studies should investigate whether these pricing dynamics hold true for other surgical oncologic procedures.

**Conclusions**

In conclusion, the price of radical bone tumor resection in the United States shows significant, often counterintuitive variability and may be driven in part by market dynamics. These findings underscore the urgent need for more granular data to understand these price drivers, which is essential for developing a more rational, predictable, and equitable pricing system for vital cancer care.

**Acknowledgments**

The authors have no conflicts of interest to disclose, and the abstract has not been submitted for consideration elsewhere.

**References**

1.	Bartelstein MK, Boland PJ. Fifty years of bone tumors. J Surg Oncol. 2022;126(5):906-12. doi: 10.1002/jso.27027. PubMed PMID: 36087079; PubMed Central PMCID: PMCPMC9524618.

2.	Gazendam A, Ghert M. What's New in Musculoskeletal Tumor Surgery. J Bone Joint Surg Am. 2023;105(24):1929-36. Epub 20231024. doi: 10.2106/JBJS.23.00833. PubMed PMID: 37874888.

3.	Errani C, Mavrogenis AF, Tsukamoto S. What's new in musculoskeletal oncology. BMC Musculoskelet Disord. 2021;22(1):704. Epub 20210817. doi: 10.1186/s12891-021-04590-1. PubMed PMID: 34404379; PubMed Central PMCID: PMCPMC8369444.

4.	Meltzer PS, Helman LJ. New Horizons in the Treatment of Osteosarcoma. N Engl J Med. 2021;385(22):2066-76. doi: 10.1056/NEJMra2103423. PubMed PMID: 34818481.

5.	Ryan C, Stoltzfus KC, Horn S, Chen H, Louie AV, Lehrer EJ, et al. Epidemiology of bone metastases. Bone. 2022;158:115783. Epub 20201201. doi: 10.1016/j.bone.2020.115783. PubMed PMID: 33276151.

6.	Franchi A. Epidemiology and classification of bone tumors. Clin Cases Miner Bone Metab. 2012;9(2):92-5. Epub 20120930. PubMed PMID: 23087718; PubMed Central PMCID: PMCPMC3476517.

7.	Schulman KL, Kohles J. Economic burden of metastatic bone disease in the U.S. Cancer. 2007;109(11):2334-42. doi: 10.1002/cncr.22678. PubMed PMID: 17450591.

8.	Sonkin D, Thomas A, Teicher BA. Cancer treatments: Past, present, and future. Cancer Genet. 2024;286-287:18-24. Epub 20240617. doi: 10.1016/j.cancergen.2024.06.002. PubMed PMID: 38909530; PubMed Central PMCID: PMCPMC11338712.

9.	Blasius F, Delbruck H, Hildebrand F, Hofmann UK. Surgical Treatment of Bone Sarcoma. Cancers (Basel). 2022;14(11). Epub 20220529. doi: 10.3390/cancers14112694. PubMed PMID: 35681674; PubMed Central PMCID: PMCPMC9179414.

10.	Hayashi K, Tsuchiya H. The role of surgery in the treatment of metastatic bone tumor. Int J Clin Oncol. 2022;27(8):1238-46. Epub 20220228. doi: 10.1007/s10147-022-02144-6. PubMed PMID: 35226235.

11.	Xu M, Wang Z, Yu XC, Lin JH, Hu YC. Guideline for Limb-Salvage Treatment of Osteosarcoma. Orthop Surg. 2020;12(4):1021-9. Epub 20200706. doi: 10.1111/os.12702. PubMed PMID: 32633103; PubMed Central PMCID: PMCPMC7454155.

12.	Kutzer KM, Kiwinda LV, Yang D, Mitchell JK, Luo EJ, Harman EJ, et al. Insurance Payor Status and Outcomes in Pediatric Sports-Related Injuries: A Rapid Review. Clin Pract. 2025;15(3). Epub 20250304. doi: 10.3390/clinpract15030052. PubMed PMID: 40136588; PubMed Central PMCID: PMCPMC11940847.

13.	Kutzer KM, Wu KA, Shenoy D, Therien A, Shah SN, Ryan S, et al. State Healthcare Regulations and Political Affiliation Associated with Total Hip Arthroplasty Prices Across the United States. J Arthroplasty. 2025. Epub 20250411. doi: 10.1016/j.arth.2025.04.007. PubMed PMID: 40222431.

14.	Wu KA, Baker RL, Hurley ET, Kutzer KM, Choudhury A, Pean CA, et al. Health care policy and arthroscopic rotator cuff repair prices in the United States. J Shoulder Elbow Surg. 2025;34(1):368-74. Epub 20240919. doi: 10.1016/j.jse.2024.07.043. PubMed PMID: 39303900.

15.	Wu KA, Kutzer KM, Doyle TR, Hurley ET, Pean CA, Anakwenze O, et al. The impact of political partisanship, certificate of need, Medicaid expansion, and area deprivation index on total shoulder arthroplasty prices in the United States. J Shoulder Elbow Surg. 2025;34(1):361-7. Epub 20240730. doi: 10.1016/j.jse.2024.05.051. PubMed PMID: 39084406.

16.	Kasthuri VS, Alsoof D, Balmaceno-Criss M, Daher M, McDonald CL, Diebo BG, et al. Variability in expenses related to spine oncology care: comparison of payer-negotiated rates at National Cancer Institute-Designated Cancer Centers. Spine J. 2024;24(2):304-9. Epub 20231029. doi: 10.1016/j.spinee.2023.10.008. PubMed PMID: 38440969.

17.	Sankaran R, O'Connor J, Nuliyalu U, Diaz A, Nathan H. Payer-Negotiated Price Variation and Relationship to Surgical Outcomes for the Most Common Cancers at NCI-Designated Cancer Centers. Ann Surg Oncol. 2024;31(7):4339-48. Epub 20240320. doi: 10.1245/s10434-024-15150-x. PubMed PMID: 38506934.

18.	Hospital Price Transparency Rule: Centers for Medicare & Medicaid Services; 2021 [cited 2025 March 7]. Available from: <https://www.cms.gov/priorities/key-initiatives/hospital-price-transparency>.

19.	Shenoy DA, Therien AD, Poehlein E, Zirbes C, Kutzer K, Wu KA, et al. Hospital and Regional-Level Factors Associated with Negotiated Payor Rates for Hip Fracture Fixation: A Multistate Analysis. Value Health. 2025. Epub 20250930. doi: 10.1016/j.jval.2025.09.3051. PubMed PMID: 41038447.

20.	Clear Rates Data: Price Transparency Data at Your Fingertips.: Turquoise Health; [cited 2024 <https://turquoise.health/products/clear_rates_data>]. Available from: <https://turquoise.health/>.

21.	Status of State Action on the Medicaid Expansion Decision: Kaiser Family Foundation; 2024 [cited 2025]. Available from: <https://www.kff.org/affordable-care-act/state-indicator/state-activity-around-expanding-medicaid-under-the-affordable-care-act/?currentTimeframe=0&sortModel=%7B%22colId%22:%22Location%22,%22sort%22:%22asc%22%7D>.

22.	Certificate of Need State Laws: National Conference of State Legislatures; 2024 [cited 2025 June 29]. Available from: <https://www.ncsl.org/health/certificate-of-need-state-laws>.

23.	Hollenbeck BK, Kaufman SR, Oerline M, Modi PK, Caram MEV, Shahinian VB, et al. Effects of Advanced Practice Providers on Single-specialty Surgical Practice. Ann Surg. 2023;277(1):e40-e5. Epub 20210303. doi: 10.1097/SLA.0000000000004846. PubMed PMID: 33914476; PubMed Central PMCID: PMCPMC8989058.

24.	State Practice Environment: American Association of Nurse Practitioners; 2024 [cited 2025]. Available from: <https://www.aanp.org/advocacy/state/state-practice-environment>.

25.	All-Payer Claims Database Legislation by State Institute for Health Policy and Practice (IHPP) at the University of New Hampshire (UNH): National Association of Health Data Organizations (NAHDO); 2024 [cited 2025]. Available from: <https://www.apcdcouncil.org/state-efforts/apcd-legislation-state>.

26.	Joynt KE, Harris Y, Orav EJ, Jha AK. Quality of care and patient outcomes in critical access rural hospitals. JAMA. 2011;306(1):45-52. doi: 10.1001/jama.2011.902. PubMed PMID: 21730240; PubMed Central PMCID: PMCPMC3337777.

27.	Census Regions and Divisions of the United States: United States Census Bureau; 2025 [cited 2025 October 1]. Available from: <https://www2.census.gov/geo/pdfs/maps-data/maps/reference/us_regdiv.pdf>.

28.	White C, Reschovsky JD, Bond AM. Understanding differences between high- and low-price hospitals: implications for efforts to rein in costs. Health Aff (Millwood). 2014;33(2):324-31. Epub 20140129. doi: 10.1377/hlthaff.2013.0747. PubMed PMID: 24476706.

29.	Beaulieu ND, Chernew ME, McWilliams JM, Landrum MB, Dalton M, Gu AY, et al. Organization and Performance of US Health Systems. JAMA. 2023;329(4):325-35. doi: 10.1001/jama.2022.24032. PubMed PMID: 36692555.

30.	Schaefer SL, Ibrahim AM. Overcoming Geographic Barriers: Surgical Care in Rural Populations. Clin Colon Rectal Surg. 2025;38(1):41-8. Epub 20240425. doi: 10.1055/s-0044-1786390. PubMed PMID: 39734717; PubMed Central PMCID: PMCPMC11679254.

31.	Henderson AP, Moore ML, Holle AM, Haglin JM, Brinkman JC, Van Schuyver PR, et al. Medicaid Reimbursement for Total Hip and Knee Arthroplasty: A State-by-State Analysis Compared With Medicare. J Arthroplasty. 2025;40(2):320-7. Epub 20240822. doi: 10.1016/j.arth.2024.08.021. PubMed PMID: 39178973.

32.	Moore ML, Henderson A, Haglin JM, Brinkman JC, Van Schuyver PR, Bingham JS, et al. Medicaid Reimbursement for Common Orthopaedic Trauma Procedures Is 16.0% Less Compared With Medicare With Substantial Variability Between States. J Orthop Trauma. 2024;38(11):e362-e70. doi: 10.1097/BOT.0000000000002901. PubMed PMID: 39141357.

33.	Rallo MS, Berke CN, Shaw TC, Bunch JA, Menger RP. State-to-State Variation in Medicaid Reimbursement for Neurosurgical Procedures: Implications for Healthcare Access in Vulnerable Populations. Neurosurgery. 2024. Epub 20240119. doi: 10.1227/neu.0000000000002823. PubMed PMID: 38240565.

34.	Ejaz A, Spolverato G, Bridges JF, Amini N, Kim Y, Pawlik TM. Choosing a cancer surgeon: analyzing factors in patient decision making using a best-worst scaling methodology. Ann Surg Oncol. 2014;21(12):3732-8. Epub 20140604. doi: 10.1245/s10434-014-3819-y. PubMed PMID: 24895113.

35.	Voda AI, Bostan I. Public Health Care Financing and the Costs of Cancer Care: A Cross-National Analysis. Cancers (Basel). 2018;10(4). Epub 20180412. doi: 10.3390/cancers10040117. PubMed PMID: 29649115; PubMed Central PMCID: PMCPMC5923372.

36.	Shih YC, Ganz PA, Aberle D, Abernethy A, Bekelman J, Brawley O, et al. Delivering high-quality and affordable care throughout the cancer care continuum. J Clin Oncol. 2013;31(32):4151-7. Epub 20131014. doi: 10.1200/JCO.2013.51.0651. PubMed PMID: 24127450; PubMed Central PMCID: PMCPMC3816960.

37.	Niraula S. Strategizing health technology assessment for containment of cancer drug costs in a universal health care system: Case of the pan-Canadian Oncology Drug Review. Cancer. 2019;125(18):3100-3. Epub 20190602. doi: 10.1002/cncr.32209. PubMed PMID: 31154671.

38.	Longo CJ, Fitch MI, Banfield L, Hanly P, Yabroff KR, Sharp L. Financial toxicity associated with a cancer diagnosis in publicly funded healthcare countries: a systematic review. Support Care Cancer. 2020;28(10):4645-65. Epub 20200711. doi: 10.1007/s00520-020-05620-9. PubMed PMID: 32653957.

39.	Fitch MI, Longo CJ, Chan RJ. Cancer patients' perspectives on financial burden in a universal healthcare system: Analysis of qualitative data from participants from 20 provincial cancer centers in Canada. Patient Educ Couns. 2021;104(4):903-10. Epub 20200813. doi: 10.1016/j.pec.2020.08.013. PubMed PMID: 32843264.
