# Supplementary material for: Exploring the drivers of price variation in orthopaedic radical bone tumor resection: A nationwide database study
Source: PLoS One. 2026 Feb 26;21(2):e0343676. doi: 10.1371/journal.pone.0343676 (PMC12944713; doi:10.1371/journal.pone.0343676)
Supplement: Attachment — Submitted filename: R2_ReviewerResponse_PLOSOne.docx [file pone.0343676.s009.docx]

**PONE-D-25-44873R1**

**Exploring the Drivers of Price Variation in Orthopaedic Radical Bone Tumor Resection: A Nationwide Database Study**

We sincerely thank the reviewers for both their positive feedback and their suggestions for improvement. Below, we provide our point-by-point response. Please note that the line numbers provided correspond to the “Tracked Changes” version of the manuscript.

**Reviewer #4, Comment #1**: Manuscript is now suitable for acceptance. The authors have clearly addressed the major issues raised, and the manuscript is now suitable for acceptance.

- **Author Response**: Thank you for your positive assessment. We hope the changes that have been made in response to Reviewer #5 now make the manuscript suitable for acceptance.

**Reviewer #5, Comment #1**: Sincere gratitude to the authors for the novel manuscript on radical resection. Although the comments earlier raised have been addressed, I still believe some revisions can still be made to make the manuscript a more robust read for the viewers. The study is mainly focused on orthopaedic surgeries thus the title be revised to reflect it. The present title may confuse readers to think of the study as being generalized to all radical resections in the whole body.

- **Author Response**: Thank you for the positive overall assessment, and feedback on our title. The title has now been revised to include the term “Orthopaedic.” (Page 1).

**Reviewer #5, Comment #2**: A list of abbreviated terms should be included in the manuscript.

- **Author Response**: We have now included a list of abbreviated terms, presented in the order that they appear in our manuscript, following the keywords section (Page 2).

**Reviewer #5, Comment #3**: Abstract. Write the full iteration of NP on Line 40.

- **Author Response**: Thank you for noting this omission. We have now spelled out NP at its first mention in the abstract.

**Reviewer #5, Comment #4**: Introduction. Recast statement as it is too long on Lines 54-57. “While primary bone cancers account for only 0.2% of primary malignancies in the U.S.( this statement should be cited accordingly (Lines 54-55))”

- **Author Response**: We have now rephrased this sentence to reduce its length. We have also made sure that the citations are provided accordingly.
- **Change(s)**:
  - “While primary bone cancers account for only 0.2% of primary malignancies in the United States (U.S.), approximately 5.1% of patients with other malignancies report developing bone metastases.[5, 6] The national cost burden for patients with metastatic bone disease estimated at $12.6 billion dollars in 2007.[7]” (Introduction, Lines 67-69).

**Reviewer #5, Comment #5**: Despite advancements in medical therapies (Line 58); this statement should be cited accordingly

- **Author Response:** Thank you for noting this opportunity for improvement. We have now cited a study that discusses advancements in sarcoma systemic therapies.

**Reviewer #5, Comment #6**: Methods. Line 103 : Following studies with similar methodologies; the studies should be cited

- **Author Response:** Thank you for noting this opportunity for improvement. We have now cited the studies with “similar methodologies” referenced in this section.

**Reviewer #5, Comment #7**: Lines 103 - 104 - "we then excluded outliers within the top and bottom 10% of all negotiated rates" ; Was this discussed with your statistician as there are statistical ways to include or exclude outliers?

- **Author Response**: We appreciate the reviewers’ attention to detail. We confirm that this decision was discussed with our statistician, and used to be consistent with studies with similar methodologies and to reduce the impact of extreme reporting artifacts. In response to Reviewer #5 Comment #6, we have now added the citations for the similar methodologies referenced.

**Reviewer #5, Comment #8**: Line 117 - Data on NP independent; Write the full meaning of NP as this is its first usage

- **Author Response**: Thank you for noting this omission. We have now added this abbreviation.

**Reviewer #5, Comment #9**: Line 127 - Procedure rates are standardized in 2024 U.S. Dollars. ; Kindly clarify 2024 US Dollars.

- **Author Response**: We have ensured that we clarify this is 2024 United States (U.S.) Dollars. We have now provided adjusted the introduction to spell out “United States” at its first mention.
  - **Added Text**: “While primary bone cancers account for only 0.2% of primary malignancies in the **United States (U.S.)…”** (Introduction, Line 67)

**Reviewer #5, Comment #10**: Line 129 - What does authors mean by bed range, does it mean the number of available beds in a facility? Authors should be very specific in language utilization.

- **Author Response**: Thank you for this comment. We have now provided a definition of total bed range.
- **Added Text:**
  - “Total bed range was used as a proxy for hospital size and refers to the approximate number of inpatient hospital beds.” (Methods, Lines 141-142)

**Reviewer #5, Comment #11**: Statistical Analysis. Line 148 - Authors should indicate that mean (SD) or median (IQR) will be used as indicated.

- **Author Response**: We have now clarified the descriptive statistics section of our methods.
- **Added Text**:
  - “First, a descriptive analysis was conducted to summarize cohort characteristics using frequencies and percentages for categorical variables, and means with standard deviation (SD) or medians with interquartile range (IQR) for continuous variables.” (Methods, Lines 162-163)

**Reviewer #5, Comment #12**: Inferential tests should also be performed across categories to glean reasonable insights into significant differences across meaningful categories.

- **Author Response**: While we appreciate the reviewer’s suggestion to perform inferential tests across categories in Table 1, we believe our current multivariate linear regression model already addresses this need in a more statistically rigorous manner. Each regression coefficient (“Estimate”) and its associated p-value represent an inferential test comparing that specific category to the reference group while also controlling for confounders (other hospital, payor, and policy-level variables). This approach avoids the increased risk of Type I errors (false positives) associated with multiple independent hypothesis tests and ensures that the differences reported are not due to confounding factors. Additionally, given that the purpose of the study was not to compare negotiated payor rates or characteristics between the two CPT codes, but rather to provide two examples of CPT codes for bone tumor resections and evaluate them separately, we did not perform inferential testing in Table 1. Therefore, Table 1 is intended purely as a descriptive cohort summary.

**Reviewer #5, Comment #13**: Authors should specify the assumptions taken to use multivariate linear regression and the parameters used to determine the variables to be included in the model. Did authors check assumptions like multicollinearity, heteroskedascity and others?

- **Author Response**: We thank the reviewer for this comment. We have updated the Statistical Analysis section to explicitly state the assumptions tested for our multivariate linear regression models. Specifically, we confirmed that our data met the requirements for linearity and normality of residuals. To address the reviewer’s concern regarding multicollinearity, we calculated Variance Inflation Factors (VIF) for all predictors, with all values remaining below a threshold of 5, indicating no significant multicollinearity.

**Reviewer #5, Comment #14**: What were the model performance metrics used to justify the validity of each of the models such as R squared or adjusted R squared? All these parameters will determine the validity of the model and the validity of the results.

**Reviewer #5, Comment #17**: Model metrics should be added to the footnotes of all models

- **Author Response**: We have now added R^2^ to the footnotes of all the regression tables, which range between 0.19-0.24. This range indicates that the predictors in our model have moderate explanatory power, aligning with our discussion of many factors that contribute to variation in negotiated prices for radical bone tumor resections. These values indicate both that while our selected variables, as expected, do not account for most of the variation, they do explain some of the variation. It also supports the complexity of the pricing market as described in our discussion section.

**Reviewer #5, Comment #15**: Line 166 - Authors should only report the statistically relevant descriptive statistic should it be median (IQR) or mean (SD). Reporting both is unnecessary.

- **Author Response**: Thank you for this comment. Given the size of our dataset, we have now removed the median (IQR) and focus solely on reporting the means with standard deviations.

**Reviewer #5, Comment #16**: the results were mostly repetition of the information on the tables and this should not be so.

- **Author Response**: Thank you for this important point. We have now significantly revised the results to remove numbers alone within the text and focused on synthesizing the data to allow for an easier read of the results section and a more direct answer to the research question. In response to comment #17, we have also replaced the regression tables with Figures and moved all tables to an eAppendix, which we also believe improves the flow and readability of the results. Consistent with guidelines^[1]^ for writing peer-reviewed manuscripts, we have not provided any interpretation of findings.
- **Added Text (examples)**:
  - “Compared to hospitals with 1-100 beds, rates mostly increased with bed count and were the highest in facilities with 1500+ beds (+$797.46, p<0.001).” (Results, Lines 206-208)
  - “Payor class was also associated with price variation.” (Results, Line 217)
  - “All payor classes had significantly lower rates when compared to the Commercial payor class, with the Medicare advantage class having the lowest rates ($-2946.11, p<0.001).” (Results, Lines 270-272)

**Reviewer #5, Comment #17**: there was over-utilization of tables; visualisation graphics such as bar plots, box plots and other visualisation means ought to be utilised.

- regression tables can be presented in more explanatory formats with importance placed on models and parameters that explain most of the variance seen in individual models

- **Author Response**: We thank the reviewer for providing this area for improvement. We have now replaced all the in-text regression tables with Figures (Figures 1-8). The Tables have now been moved as eTables to a supplementary document/Appendix.

**Reviewer #5, Comment #18**: There should be consistency in font type, size and line spacing with other sections of the manuscript.

- **Author Response**: Thank you for noting this inconsistency. We have now ensured the entire manuscript is 12-point font and double spaced, including the references.

1. https://pmc.ncbi.nlm.nih.gov/articles/PMC8520870/
